# Pilot Evaluation of Simplified HBV DNA Extraction–LAMP Workflows for Hepatitis B Molecular Testing in Resource-Limited Settings

**DOI:** 10.3390/diagnostics16172756

**Published:** 2026-08-28

**Authors:** Nang Kham-Kjing, Warit Kongfu, Patcharaporn Matthanawut, Nachcharee Srisupattanakul, Sayamon Hongjaisee, Nicole Ngo-Giang-Huong, Woottichai Khamduang

**Affiliations:** 1 Department of Medical Technology, Faculty of Associated Medical Sciences, Chiang Mai University, Chiang Mai 50200, Thailand; khamkjing.kk@gmail.com (N.K.-K.); waritkongfu.official@gmail.com (W.K.); patcharaporn.matth@gmail.com (P.M.); nachcharee88@gmail.com (N.S.); 2 LUCENT International Collaboration, Faculty of Associated Medical Sciences, Chiang Mai University, Chiang Mai 50200, Thailand; sayamon.ho@cmu.ac.th; 3 School of Health Sciences Research, Research Institute for Health Sciences, Chiang Mai University, Chiang Mai 50200, Thailand

**Keywords:** HBV, hepatitis B virus, LAMP, HBV LAMP, simple extraction

## Abstract

**Background/Objectives:** Access to hepatitis B virus (HBV) DNA testing remains limited in resource-limited settings. The main reasons for this are the high cost of molecular assays and the need for specialized infrastructure. This pilot study aimed to preliminarily develop and evaluate simple HBV DNA extraction methods combined with the colorimetric loop-mediated isothermal amplification (LAMP) technique. **Methods:** Three extraction methods were evaluated: (i) boiling-based, (ii) paper-based, and (iii) an adapted magnetic bead-based extraction method. Preliminary analytical performance was assessed using serially diluted HBV DNA-positive pooled plasma. Detection performance was evaluated using 40 clinical plasma specimens, including 35 HBV DNA-positive and 5 HBV DNA-negative samples, with real-time PCR as the reference standard. **Results:** The preliminary analytical detection level observed under the tested conditions was 10^5^ IU/mL for the LAMP methods using boiling- or paper-based extraction, while it was 10^3^ IU/mL with the adapted magnetic bead-based extraction–LAMP method. Evaluation using clinical specimens showed sensitivities of 43%, 54%, and 63%, respectively. Detection was dependent on viral load, with the highest detection observed among specimens with HBV DNA concentrations above 10^5^ IU/mL. **Conclusions:** In this laboratory pilot, the workflows showed viral-load-dependent qualitative detection, with the adapted magnetic bead-based workflow yielding the highest observed detection rate. These findings require confirmation through formal analytical validation and larger, independent clinical studies before any clinical or implementation use can be considered.

## 1. Introduction

Hepatitis B virus (HBV) is a hepatotropic DNA virus that can cause both acute and chronic liver disease. Infection may remain asymptomatic for many years but can progress to chronic hepatitis, cirrhosis, liver failure, and hepatocellular carcinoma [1]. Hepatitis B virus (HBV) infection remains a major global public health concern with a disproportionate burden borne by people living in low- and middle-income countries [2,3]. In 2024, the World Health Organization (WHO) estimated that 240 million people were living with hepatitis B worldwide [2]. However, only 27% of individuals with chronic HBV infection had been diagnosed, and less than 5% were on treatment [2]. These figures are far below the WHO targets set up in 2016 to eliminate viral hepatitis as a public health threat by 2030, i.e., diagnosing 90% of people living with chronic HBV infection and treating 80% of those who are eligible for treatment. Therefore, scaling up treatment for people with chronic HBV infection is a major priority to achieve the 2030 global viral hepatitis elimination target. However, many infected individuals are unaware of their infection status and therefore do not receive timely care and treatment [4,5].

Serological assays, including detecting hepatitis B surface antigen, are widely used for screening and identifying individuals with a current infection [6,7]. Hepatitis B e antigen (HBeAg) has been used as a surrogate marker of viral replication; however, current guidelines from the WHO, the American Association for the Study of Liver Diseases (AASLD), and the European Association for the Study of the Liver (EASL) recognize HBV DNA quantification as the preferred marker of viral replication and an important predictor of disease prognosis. HBV DNA measurement is critical for determining treatment eligibility, monitoring therapeutic response, and identifying cases in which serological markers may be inadequate or inconclusive [8,9,10]. Nevertheless, currently available HBV DNA testing platforms are either fully automated (Roche Cobas^®^ platforms, Hologic Panther platform, Abbott Molecular platforms, μTASWako g1, Cepheid GeneXpert platforms, etc.) or open systems based on real-time polymerase chain reaction (real-time PCR). Both approaches require sophisticated instruments, well-equipped laboratories, and trained technical staff. Major barriers to the implementation of HBV DNA testing in resource-limited settings include the high costs of molecular assays, cold-chain requirements, insufficient numbers of trained personnel, inadequate infrastructure, and reliance on centralized laboratory systems. Therefore, simple, affordable, and reliable diagnostic approaches are needed to improve HBV detection and public health surveillance in settings where conventional nucleic acid testing is not readily available.

Loop-mediated isothermal amplification (LAMP) technology has gained recognition as an alternative nucleic acid amplification method for decentralized molecular diagnosis. Unlike PCR, LAMP amplifies DNA under isothermal conditions using relatively simple heating equipment, such as a water bath, heat block, or portable incubator. LAMP also offers the advantages of rapid amplification, high analytical sensitivity, and visual or colorimetric readout options, making it suitable for point-of-care testing [11]. However, these general advantages alone do not establish the point-of-care or decentralized suitability of an HBV LAMP workflow, which also depends on the availability of a practical, reliable, and sufficiently sensitive HBV DNA extraction method.

DNA extraction is still one of the major obstacles to the implementation of molecular diagnostics in resource-limited settings. Commercial nucleic acid extraction kits are widely used because they produce relatively pure DNA that is compatible with downstream amplification assays. However, these kits often require multiple centrifugation or washing steps and specialized consumables, which can be costly and prohibitive for peripheral laboratories. Automated extraction platforms further improve throughput and standardization, but despite recent advances in compact and user-friendly systems, their cost and operational requirements can remain challenging for implementation in decentralized and resource-limited settings. In contrast, simplified extraction methods, such as heat treatment, crude lysis, detergent-based lysis, alkaline treatment, or low-cost precipitation approaches, may reduce costs, processing time, and dependency on equipment [12,13,14]. Such methods are especially relevant for DNA viruses such as HBV, whose target nucleic acid is more stable than RNA targets. Nevertheless, simplified extraction methods must be carefully evaluated because crude lysates may contain inhibitors and incomplete viral disruption may reduce sensitivity. Excessive sample dilution may also compromise detection at low viral loads.

In this pilot study, we aimed to (1) develop and preliminarily validate simple HBV DNA extraction methods combined with colorimetric LAMP, (2) preliminarily assess the analytical detection performance of each extraction–LAMP workflow, and (3) describe their detection performance using clinical specimens compared with real-time PCR as the reference method.

## 2. Materials and Methods

### 2.1. Study Design and Overview

This study was approved by the Human Experimentation Committee of the Research Institute for Health Sciences, Chiang Mai University, Thailand. Three extraction methods were developed and optimized: (i) a boiling-based extraction method, (ii) an adapted magnetic bead-based extraction method, and (iii) a paper-based extraction method. DNA extracts obtained from each method were subsequently tested using an HBV LAMP assay. The preliminary analytical detection level of the optimized extraction–LAMP workflows was evaluated using pooled HBV DNA-positive clinical specimens with known viral load, while preliminary detection performance was assessed using clinical specimens and compared with results obtained by real-time PCR.

### 2.2. Preparation of Pooled HBV DNA-Positive Sample 

A total of 20 leftover HBV DNA-positive plasma samples from routine diagnostic testing were randomly collected from the Clinical Microbiology Service Unit (CMSU), Faculty of Associated Medical Sciences, Chiang Mai University, Thailand. To generate a standardized HBV DNA-positive sample for method development and optimization, these samples were pooled and mixed thoroughly by vortexing. The HBV DNA viral load of the pooled sample was quantified using the Cobas HBV assay with the Cobas 6800 system (Roche Molecular Systems, Pleasanton, CA, USA) according to the manufacturer’s instructions. The pooled sample with an HBV viral load of 1.22 × 10^8^ IU/mL was aliquoted into 1 mL to minimize repeated freeze–thaw cycles and stored at −70 °C until further use. This pooled HBV DNA-positive sample was subsequently used for the initial protocol development, the optimization of DNA extraction conditions, and the determination of the preliminary analytical detection level of each extraction method when combined with LAMP.

### 2.3. Development of Simplified HBV DNA Extraction Methods

#### 2.3.1. Boiling-Based HBV DNA Extraction

To minimize reagent requirements and eliminate the need for ultracentrifugation, a boiling-based HBV DNA extraction method was developed. Briefly, 200 µL of plasma sample was incubated at 100 °C for 10 min in a dry bath incubator to release viral DNA. After heat treatment, the tube was spun down for 5 min. The supernatant containing HBV DNA was carefully transferred to a new sterile nuclease-free microcentrifuge tube without disturbing any pellet or debris.

#### 2.3.2. Paper-Based HBV DNA Extraction

To reduce the need for centrifugation or specialized equipment, a paper-based extraction method was developed. A paper strip was prepared by cutting a Whatman™ 903 Protein Saver Card (Catalog number 10534612; GE Healthcare Bio-Sciences Corp., Westborough, MA, USA) into pieces of approximately 4 mm × 70 mm. Twenty-five microliters of plasma were mixed with 25 µL of lysis buffer from Zybio Nucleic Acid Extraction Kit (Zybio Inc., Chongqing, China) and 2 µL of proteinase K. The mixture was incubated at 56 °C for 5 min to promote viral lysis and release of HBV DNA. After lysis, the prepared paper strip was immersed in the lysate and incubated at room temperature for 2 min to allow nucleic acid adsorption onto the paper matrix. Following incubation, the strip was removed using sterile forceps and washed in 50 µL of absolute ethanol for 2 min. The strip was subsequently air-dried for 15 min to completely remove the ethanol. To elute the DNA, the lower half of the strip was cut and put into a tube containing 100 µL of nuclease-free water, which was then vortexed for 15 s. The eluate was then carefully collected.

#### 2.3.3. Adapted Magnetic Bead-Based HBV DNA Extraction

Magnetic bead-based extraction was performed using a nucleic acid extraction kit (Zybio Inc., Chongqing, China) for an automated extractor by adapting the protocol into manual extraction for feasibility of use in low-resource settings. Briefly, 200 µL of plasma was mixed with 500 µL of lysis buffer and 15 µL of Proteinase K and incubated at 55 °C for 4 min. Following lysis, 150 µL of magnetic bead suspension was added to the lysate and mixed by pipetting. The mixture was incubated at room temperature for 5 min to allow DNA binding to the magnetic beads. Then, the tube was placed on a magnetic rack (Macherey-Nagel, Düren, Germany) until the solution became clear. The supernatant was carefully removed without disturbing the bead pellet. The beads were washed twice with 400 µL of wash buffer. Each wash step was performed by resuspending the beads thoroughly and collecting them again using the magnetic rack. After the final wash, the beads were air-dried for approximately 2 min at room temperature to remove residual ethanol or wash buffer. DNA was eluted by adding 60 µL of elution buffer to the dried beads. The mixture was then incubated at 80 °C for 2 min. The tube was then placed on the magnetic rack, and the eluate containing purified HBV DNA was transferred to a new nuclease-free tube and stored at −70 °C until use.

### 2.4. Optimization of HBV DNA Extraction Methods

#### 2.4.1. Optimization of Boiling-Based Extraction

The boiling extraction protocol was optimized by varying the sample input volume and the inclusion of lysis buffer. The tested conditions included (i) 40 µL of plasma without lysis buffer, (ii) 40 µL of plasma mixed with 200 µL of lysis buffer, (iii) 200 µL of plasma without lysis buffer, and (iv) 200 µL of plasma mixed with 1 mL of lysis buffer. All samples were incubated at 100 °C for 10 min and then spun down for 5 min to collect the supernatant. The extracted DNA concentration was measured using Qubit dsDNA HS Assay Kits (Thermo Fisher Scientific, Eugene, OR, USA). The optimal condition was selected based on DNA yield and LAMP positivity.

#### 2.4.2. Optimization of Paper-Based Extraction

To optimize the paper-based DNA extraction method, different washing reagents and elution strategies were evaluated. Briefly, 25 µL of plasma was mixed with 25 µL of lysis buffer from the Zybio Nucleic Acid Extraction Kit (Zybio Inc., Chongqing, China) and 2 µL of proteinase K. The mixture was incubated at 56 °C for 5 min. A paper strip was then immersed in the lysate for 2 min and subsequently transferred to a separate wash tube containing either 50 µL of kit-provided wash buffer or 50 µL of absolute ethanol and incubated for 15 min. After the washing step, the strip was placed on parafilm and air-dried at room temperature for approximately 15 min until completely dry. Two downstream processing conditions were evaluated. In the first condition (direct strip-to-LAMP), the dried strip was cut and transferred directly into the LAMP reaction mixture without elution. In the second condition (eluate-to-LAMP), the lower part of the dried strip was excised and incubated in 100 µL of nuclease-free water to elute nucleic acids, which were then used as the template for the LAMP assay.

### 2.5. HBV Colorimetric LAMP Assay Based on Cresol Red

For LAMP, primers targeting the HBV S and polymerase genes (nucleotides 228–421) designed by a previous study were used [15]. The extracted DNA was amplified using a colorimetric LAMP assay as described in our previous study [16]. Briefly, a total volume of 25 µL reaction mixture containing 10 mM ammonium sulfate (NH_4_)_2_SO_4_, 50 mM potassium chloride (KCl), 0.1% (*v*/*v*) Tween-20, 8 mM magnesium sulfate (MgSO_4_), and 1.4 mM dNTP mix was prepared. The pH was adjusted to a range of 8–9 using 1 M potassium hydroxide (KOH). Then, the reaction was mixed with 1.2 µM each of forward inner (FIP) and reverse inner (RIP) primers, 0.8 µM each of loop-forward (LF) and loop-reverse (LR) primers, 0.4 µM each of forward (F3) and reverse (R3) primers, 8 U of *Bst* DNA polymerase (New England Biolabs, Ipswich, MA, USA), 100 µM of cresol red (Merck, Darmstadt, Germany), and 2 µL of template. LAMP was performed at 65 °C for 50 min and inactivated at 80 °C for 10 min. Positive and negative controls were included in each run. The positive control consisted of known HBV DNA-positive extract, while the negative control (non-template control, NTC) consisted of nuclease-free water. The release of protons during the amplification process led to a decrease in the pH level, resulting in a change in cresol red color from pink to yellow, while a negative result remained pink. Gel electrophoresis was used to confirm the results with a ladder-like banding pattern indicating positive amplification. To minimize carry-over contamination, pre- and post-amplification procedures were performed in separate rooms using filtered pipette tips. Work surfaces and the biosafety cabinet were disinfected with 70% ethanol and exposed to UV irradiation before and after each experiment. Each LAMP run included a positive control and NTC to monitor assay performance and potential contamination.

### 2.6. Preliminary Analytical Detection Level Assessment

To evaluate the preliminary analytical detection level of each extraction–LAMP workflow, the quantified pooled HBV DNA-positive sample was serially diluted in normal human plasma. Ten-fold serial dilutions were prepared to obtain HBV DNA concentrations ranging from 10^6^ to 10^1^ IU/mL. The same dilution series was used for boiling-based extraction, adapted magnetic bead-based extraction, and paper-based extraction. Each dilution was processed using the respective developed extraction protocols followed by LAMP. Both the extraction and amplification reactions were carried out in triplicate.

### 2.7. Clinical Evaluation of Extraction Methods

To further evaluate the preliminary detection performance of each extraction–LAMP workflow, a total of 35 leftover clinical samples positive for HBV DNA with different viral loads were retrieved, together with 5 normal human plasma samples to serve as negative samples. Specifically, 5 samples were selected from each HBV viral load category: <2.00, 2.01–3.00, 3.01–4.00, 4.01–5.00, 5.01–6.00, 6.01–7.00, and 7.01–8.00 log_10_ IU/mL. The HBV viral load for each sample is presented in Table S1. Each clinical sample was extracted using the developed boiling-based, adapted magnetic bead-based, and paper-based extraction methods. Extracted DNA from each method was then tested using the HBV LAMP assay, and the results were confirmed using gel electrophoresis. The LAMP results were interpreted independently and compared with the corresponding real-time PCR results. The real-time PCR results were used as the reference standard for the analysis of detection performance. For clinical interpretation, HBV DNA-positive samples were additionally categorized according to clinically relevant HBV DNA thresholds (<2000, 2000–20,000, and >20,000 IU/mL), and the detection rates for each extraction–LAMP workflow were compared across these categories.

### 2.8. Data Analysis

The binary variable results between LAMP and real-time PCR were used to calculate specificity, sensitivity, positive predictive value (PPV), and negative predictive value (NPV). The 95% confidence intervals (95% CI) for the proportion were calculated. Statistical analyses were performed using RStudio version 4.4.2 (RStudio, Boston, MA, USA).

### 2.9. Ethics Statement

This study was conducted in accordance with the Declaration of Helsinki and was approved by the Human Experimentation Committee of the Research Institute for Health Sciences, Chiang Mai University, Thailand (approval No. 56/2024, study code No. 30/2024) on 29 August 2024. This study involved the secondary use of fully de-identified stored plasma samples obtained from routine care. Because all samples were anonymized before analysis and no personally identifiable information was used, informed consent was not required in accordance with institutional regulations. 

## 3. Results

### 3.1. Optimization of HBV DNA Extraction Conditions

**Figure 1:**
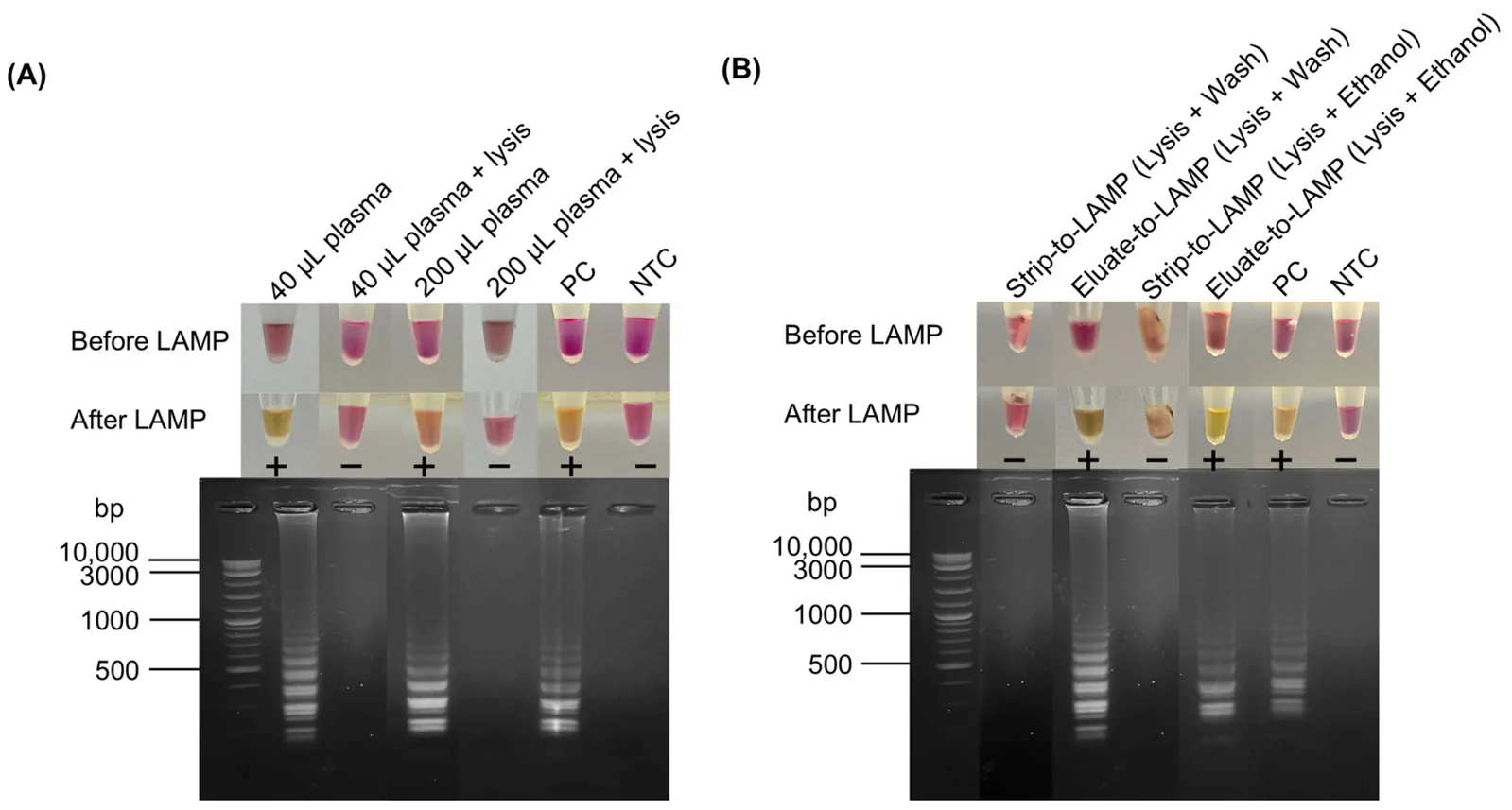
Optimization of simplified HBV DNA extraction methods for colorimetric LAMP detection. (**A**) Optimization of the boiling-based HBV DNA extraction method. Four boiling-based extraction conditions were evaluated before and after LAMP using cresol red colorimetric detection, followed by confirmation with gel electrophoresis. Lane 1: 40 µL plasma; Lane 2: 40 µL plasma with lysis buffer; Lane 3: 200 µL plasma; Lane 4: 200 µL plasma with lysis buffer. Positive LAMP reactions were indicated by a color change from pink to yellow and by the presence of a ladder-like banding pattern on gel electrophoresis. Negative reactions remained pink and showed no amplification bands. (**B**) Optimization of the paper-based HBV DNA extraction method. Four paper-based extraction conditions were evaluated using lysis buffer combined with different washing and elution strategies. Lane 1: Lysis buffer followed by wash buffer washing and direct strip-to-LAMP reaction. Lane 2: Lysis buffer followed by wash buffer washing and eluate-to-LAMP reaction. Lane 3: Lysis buffer followed by absolute ethanol washing and direct strip-to-LAMP reaction. Lane 4: Lysis buffer followed by absolute ethanol washing and eluate-to-LAMP reaction; PC, positive control; NTC, non-template control; bp, base pairs.

Optimization steps were performed using a standardized pooled HBV DNA-positive plasma sample with a viral load of 1.22 × 10^8^ IU/mL. Using the boiling-based extraction method, direct boiling of 200 µL plasma produced a higher DNA concentration than boiling 40 µL plasma (0.5 ng/µL and 0.2 ng/µL, respectively) and generated positive HBV LAMP results, as demonstrated by a visible color change from pink to yellow and the presence of a characteristic ladder-like amplification pattern on gel electrophoresis (Figure 1A). In contrast, boiling in the presence of lysis buffer yielded higher DNA concentrations: 160.9 ng/µL for 200 µL of plasma and 2.4 ng/µL for 40 µL of plasma. However, these lysates tested negative by HBV LAMP, with no visible color change and no amplification band observed on gel electrophoresis. Based on combined DNA yield and amplification compatibility, direct boiling of 200 µL plasma without lysis buffer was selected as the optimized boiling-based extraction condition for subsequent experiments.

Using the paper-based extraction method, both wash buffer-based and ethanol-based washing followed by an elution step (eluate-to-LAMP reaction) produced positive LAMP results, as indicated by colorimetric amplification and confirmatory gel electrophoresis (Figure 1B). In contrast, the direct strip-to-LAMP reaction failed to amplify the target, regardless of the washing step. No color change or ladder-like pattern was observed under either direct strip-to-LAMP condition. As ethanol washing followed by an elution step provided a simple, low-cost, and LAMP-compatible workflow, this condition was selected as the optimized paper-based extraction protocol for further evaluation.

### 3.2. Preliminary Analytical Detection Performance of the Extraction–LAMP Workflows

The preliminary analytical detection performance of each extraction–LAMP workflow, determined in triplicate, is shown in Table 1. The boiling-based and paper-based extraction–LAMP workflows showed positive results in all three replicates at 10^5^ IU/mL (Figure S1A,B) and no positive results at 10^4^ IU/mL or below. The adapted magnetic bead-based extraction–LAMP workflow produced positive results in all three replicates at 10^4^ IU/mL and in two of three replicates at 10^3^ IU/mL (Figure S1C). The positive control showed successful amplification in all assays, whereas the NTC remained negative, confirming the validity of the LAMP reactions.

### 3.3. Preliminary Detection Performance in Clinical Specimens

**Figure 2:**
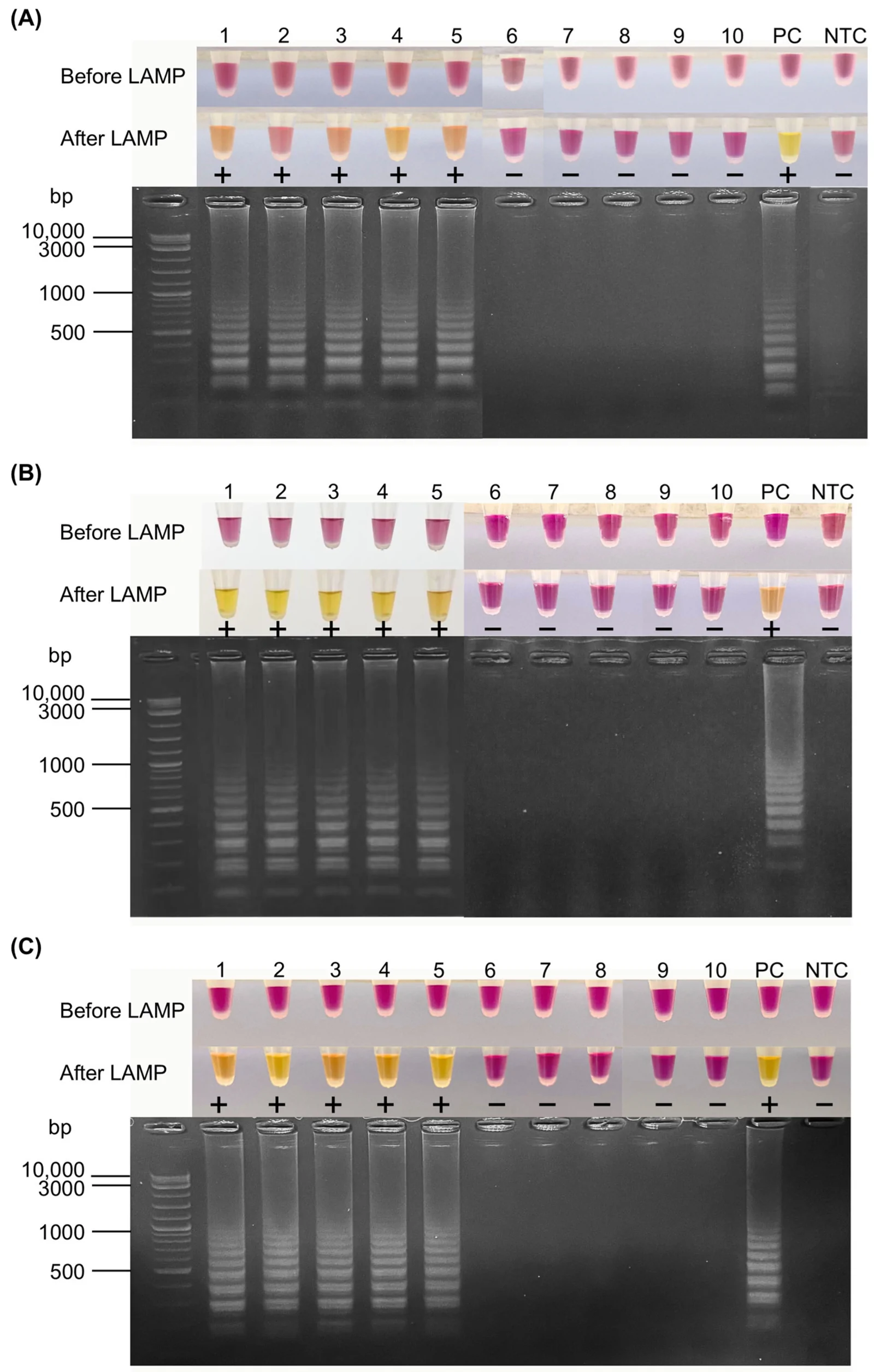
Representative clinical specimens showing preliminary detection performance of simplified HBV DNA extraction methods combined with colorimetric LAMP. Detection performance of (**A**) the boiling-based extraction–LAMP method, (**B**) the paper-based extraction–LAMP method, and (**C**) the adapted magnetic bead-based extraction–LAMP method. Lanes 1–5 represent HBV DNA-positive clinical samples, and lanes 6–10 represent HBV DNA-negative clinical samples. PC, positive control; NTC, non-template control; bp, base pairs.

Results of the preliminary detection performance of the optimized extraction methods evaluated using clinical samples are shown in Table 2. Representative results for the boiling-based, paper-based, and adapted magnetic bead-based extraction methods are shown in Figure 2. The boiling-based extraction–LAMP method detected HBV DNA in 15 of 35 positive samples, corresponding to a sensitivity of 43% (95% CI: 28–59%). This method showed a specificity of 100% (95% CI: 57–100%), positive predictive value (PPV) of 100% (95% CI: 80–100%), negative predictive value (NPV) of 20% (95% CI: 9–39%), and overall accuracy of 50% (95% CI: 35–65%). The paper-based extraction–LAMP method showed slightly improved performance, detecting 19 of 35 positive samples, with a sensitivity of 54% (95% CI: 38–70%), specificity of 100% (95% CI: 57–100%), PPV of 100% (95% CI: 83–100%), NPV of 24% (95% CI: 11–45%), and overall accuracy of 60% (95% CI: 45–74%). Among the three methods, the adapted magnetic bead-based extraction–LAMP method demonstrated the highest sensitivity, detecting 22 of 35 positive samples, with a sensitivity of 63% (95% CI: 46–77%), specificity of 100% (95% CI: 57–100%), PPV of 100% (95% CI: 85–100%), NPV of 28% (95% CI: 13–51%), and overall accuracy of 68% (95% CI: 52–80%).

### 3.4. Preliminary Detection Performance Stratified by HBV Viral Load

Detection performance of the developed extraction–LAMP methods stratified by real-time PCR viral load is shown in Table 3 and Table S1. All three extraction–LAMP workflows showed viral-load-dependent detection, with higher positivity rates among samples with higher HBV DNA levels. For the boiling-based extraction–LAMP method, detection was 100% in samples with viral loads of 6.01–8.00 log_10_ IU/mL, decreased to 80% at 5.01–6.00 log_10_ IU/mL and 20% at 4.01–5.00 log_10_ IU/mL, and was not detected below 4.00 log_10_ IU/mL. The paper-based extraction–LAMP method showed 100% detection in the 5.01–7.00 log_10_ IU/mL groups and declined at lower viral loads, with 60% positivity at 4.01–5.00 log_10_ IU/mL, 20% at 2.01–3.00 log_10_ IU/mL, and no detection below 2.00 log_10_ IU/mL or in the 3.01–4.00 log_10_ IU/mL group. The adapted magnetic bead-based extraction–LAMP method showed the best performance, with 100% detection from 5.01 to 8.00 log_10_ IU/mL, 80% at 4.01–5.00 log_10_ IU/mL, 40% at 3.01–4.00 log_10_ IU/mL, and 20% at 2.01–3.00 log_10_ IU/mL, with no detection below 2.00 log_10_ IU/mL. Based on the observed results in this pilot panel, complete detection observed from the boiling-based, paper-based, and adapted magnetic bead-based extraction–LAMP methods was 6.01–7.00 log_10_ IU/mL, 5.01–6.00 log_10_ IU/mL, and 5.01–6.00 log_10_ IU/mL, respectively.

### 3.5. Preliminary Detection Performance Stratified by Clinically Relevant HBV DNA Thresholds

To further evaluate the detection performance of the simplified extraction–LAMP workflows, detection rates were also analyzed according to clinically relevant HBV DNA thresholds (Table S2). Detection rates increased with increasing HBV DNA levels for all three extraction methods. Among the 11 samples with HBV DNA levels < 2000 IU/mL, only one sample (9%) was detected by both the paper-based and adapted magnetic bead-based extraction–LAMP methods, while no samples were detected using the boiling-based extraction–LAMP. For samples with HBV DNA levels between 2000 and 20,000 IU/mL, only the adapted magnetic bead-based extraction–LAMP method detected HBV DNA, with a detection rate of 40% (2/5). In contrast, all three extraction methods demonstrated substantially improved performance in samples with HBV DNA levels > 20,000 IU/mL. The adapted magnetic bead-based extraction–LAMP method detected all 19 samples (100%), followed by the paper-based extraction–LAMP method (18/19, 95%) and the boiling-based extraction–LAMP method (15/19, 79%).

## 4. Discussion

This laboratory pilot study provides preliminary comparative data on simplified HBV DNA extraction–LAMP workflows. Detection performance varied according to extraction strategy and HBV DNA concentration. The workflows showed clear differences in preliminary detection performance, with an analytical detection level of 10^5^ IU/mL for both boiling-based and paper-based extraction–LAMP methods and 10^3^ IU/mL for the adapted magnetic bead-based extraction–LAMP method. In preliminary evaluation using clinical specimens, no false-positive results were observed among the five HBV DNA-negative specimens included in this pilot panel, whereas the detection rate was lower and varied by method, reaching 43% for boiling-based extraction–LAMP, 54% for paper-based extraction–LAMP, and 63% for the adapted magnetic bead-based extraction–LAMP method. These findings indicate that simplified extraction–LAMP workflows may be useful for detecting moderate-to-high HBV DNA levels.

No false-positive results were observed in the small HBV DNA-negative panel evaluated in this study. However, the limited number of negative specimens resulted in a wide confidence interval and prevented reliable estimation of clinical specificity, and these findings should therefore be interpreted cautiously. Previous HBV LAMP studies have reported higher specificity and sensitivity than observed in the present study [15,17,18]. Nyan D.C. et al. reported that HBV LAMP can detect major HBV genotypes with high specificity and detected 69 of 75 HBV DNA-positive plasma samples, corresponding to 92% sensitivity [15]. Moreover, another study similarly reported 92% sensitivity and 100% specificity using HBV LAMP combined with a lateral flow device [17], while a meta-analysis also reported pooled sensitivity and specificity of 0.92 and 0.86, respectively, for LAMP-based HBV detection [18]. The absence of false-positive reactions in this small pilot panel indicated that no non-specific positive reactions were observed under the tested laboratory conditions; however, substantially larger numbers of negative specimens are required before specificity or reliability can be estimated with adequate precision. Furthermore, all positive LAMP results were accompanied by the expected ladder-like pattern on gel electrophoresis. However, clinical sensitivity in the present study was lower than that reported in several previous HBV LAMP studies. This difference does not contradict the utility of LAMP itself but rather highlights the importance of DNA extraction. Unlike many assay-development studies that use purified DNA or more efficient extraction procedures, this study intentionally evaluated simplified extraction strategies designed for potential implementation in areas with limited resources. Therefore, the reduced sensitivity likely reflects a trade-off between extraction simplicity, template recovery, inhibitor removal, and downstream amplification efficiency.

The preliminary analytical detection level results were different among the three developed extraction–LAMP methods. The boiling-based and paper-based extraction–LAMP methods showed detectable amplification down to 10^5^ IU/mL, whereas the adapted magnetic bead-based extraction–LAMP method improved analytical sensitivity to 10^3^ IU/mL. Although the analytical detection level achieved by the adapted magnetic bead-based extraction–LAMP method in the present study was higher than those reported in previous studies [19,20], it remained substantially better than those observed for the other two methods. This supports the interpretation that magnetic bead extraction improves template concentration and inhibitor removal, even when adapted into a manual, lower-resource format. In contrast, boiling and paper-based methods are less sensitive, but they require fewer resources, making them potentially useful for detection of high-viremia samples. The boiling-based method also provided the shortest turnaround time (~15 min) and lowest reagent cost, whereas the paper-based method offered a similarly low-cost alternative with only a modest increase in processing time (~25 min). In comparison, the adapted magnetic bead-based method required additional consumables, equipment, and hands-on time but achieved substantially improved analytical sensitivity. However, these approaches provide greater operational simplicity at the expense of reduced analytical sensitivity. This is consistent with evidence from HBV DNA testing using dried blood spot samples, where the choice of extraction methods and amplification protocol substantially influences detection performance [21]. Therefore, boiling and paper-based approaches could be useful for the detection of high-viremia samples.

The optimization findings further reinforce that DNA concentration alone is not sufficient for selecting extraction conditions. In the boiling-based workflow, lysis buffer-containing conditions produced higher DNA concentrations but failed to amplify by LAMP, whereas direct boiling without lysis buffer produced lower DNA concentrations but successful amplification. The presence of lysis buffer or inhibitors, which may have altered ionic strength, reaction pH, or magnesium availability, likely impairs polymerase activity, primer annealing, and efficient amplification [22,23,24]. This observation is consistent with previous reports showing that crude extraction reagents and common molecular inhibitors can affect LAMP performance by delaying amplification, reducing amplicon formation, or interfering with colorimetric detection [24,25]. These findings are particularly important for colorimetric LAMP because amplification and visual detection depend on both enzymatic activity and pH-dependent dye transition. Similarly, the paper-based workflow required an elution step before LAMP, where direct strip-to-LAMP failed despite washing with either wash buffer or ethanol. This finding indicates that, although paper-based nucleic acid capture can simplify sample processing, direct introduction of the paper matrix into the amplification reaction may inhibit LAMP or reduce template accessibility. The elution step likely improves template release and reduces the carryover of inhibitors. Among the tested conditions, ethanol washing followed by elution was selected because it provided a simple and low-cost workflow while maintaining LAMP compatibility. The use of ethanol washing and elution provided a relatively simple laboratory workflow; however, its operational feasibility, cost, reproducibility, and suitability for use outside a controlled laboratory environment were not evaluated in the present study.

When the results were stratified according to viral load, all three methods showed improved detection at higher HBV DNA levels, whereas detection declined markedly at lower viral loads. The boiling-based extraction–LAMP method showed complete detection from 6.01 to 8.00 log_10_ IU/mL, while paper-based and adapted magnetic bead-based extraction–LAMP methods showed complete detection from 5.01 log_10_ IU/mL upward. In our previous study using the commercial TIANamp Virus DNA/RNA extraction kit, the rate of HBV DNA detection among samples with a viral load of >3.0 log_10_ IU/mL was 83% and was not different from that observed by the adapted magnetic bead-based extraction method shown in this study (84%) [16]. Similarly, extraction of samples with viral loads above 4.0 log_10_ IU/mL, regardless of the extraction method used, yielded similar detection rates (90% vs. 95%). However, a direct side-by-side comparison with a commercial extraction kit was not performed in the present study. Therefore, the observed differences between extraction–LAMP workflows should be interpreted cautiously, and future studies should include simultaneous evaluation against a validated commercial extraction protocol to better distinguish extraction efficiency from amplification performance. Nevertheless, this viral-load-dependent performance is consistent with the intended role of many decentralized HBV molecular tests, which are designed to expand access to HBV DNA testing and support rapid identification of individuals with high-level viremia, rather than to replace centralized laboratory-based quantitative viral load testing [19,26,27]. This interpretation also aligns with evidence that dried blood spot HBV DNA testing performs better at higher viral loads and may be less reliable for excluding low-level viremia [28,29]. WHO evidence summaries have similarly noted that DBS-based HBV DNA testing has good sensitivity above clinically relevant thresholds but may miss samples with lower viral loads [30]. 

When stratifying the detection performance according to clinically relevant HBV DNA thresholds, the detection rate was higher among samples with HBV DNA levels > 20,000 IU/mL, particularly for the adapted magnetic bead-based workflow. According to the treatment guideline, HBV DNA thresholds of 2000 IU/mL and 20,000 IU/mL are widely used in treatment decision-making and patient management [31,32]. Although these workflows can be used to test HBV DNA in individuals with active or higher-level HBV replication, these findings are descriptive, and their limited sensitivity at lower viral loads with relatively low NPVs indicated that they should not be used as a stand-alone method to exclude HBV infection or for treatment monitoring. Larger studies with predefined clinical categories are required to determine whether the observed viral-load-dependent detection pattern is reproducible. Furthermore, successful implementation of simplified HBV molecular testing workflows will require consideration of local infrastructure, reagent supply chains, personnel training, and integration into existing referral systems. Although their clinical, decentralized, or implementation utility cannot be directly determined from the present study, these findings provide preliminary laboratory data that may inform future development and validation of simplified HBV molecular testing approaches.

This study has some limitations. First, since this is a pilot study, the clinical evaluation included a relatively small number of specimens, particularly HBV-negative samples, which limits the precision of specificity and predictive value estimates. Consequently, estimates of detection sensitivity, specificity, predictive values, and accuracy should be interpreted with caution. These estimates should not be generalized to screening or routine clinical populations, and larger studies are needed to further validate its performance. In addition, spike-in recovery experiments should be incorporated in future studies to quantitatively assess the reduction in sensitivity in each step. Furthermore, assessments of inter-day reproducibility, inter-operator variability, and formal Clinical Laboratory Standards Institute (CLSI)-style limit of detection studies should also be included in future studies. Second, the assay was qualitative and cannot provide viral load quantification, which remains essential for treatment indication and monitoring and assessment of virological suppression. Third, only plasma samples were evaluated under laboratory conditions. Further studies should assess performance using more diverse clinical sample sets, such as finger-prick blood, dried blood spots, or field-collected specimens, to confirm the generalizability of the developed methods. Nevertheless, the present findings provide a useful comparative framework showing that simplified extraction–LAMP workflows can expand access to HBV DNA detection, but the degree of simplification directly affects sensitivity. Among the three workflows evaluated in this pilot study, the adapted magnetic bead-based extraction–LAMP workflow showed the highest observed analytical and clinical-specimen detection performance and therefore represents the most appropriate candidate for further laboratory optimization and validation. However, the boiling-based and paper-based extraction–LAMP workflows showed lower detection rates and may warrant further laboratory investigation to determine whether additional optimization can improve their analytical performance.

## 5. Conclusions

This pilot study demonstrated the feasibility of simplified extraction–LAMP workflows and provided preliminary comparative data for future optimization and validation. Detection was strongly dependent on HBV DNA concentration, with the magnetic bead-based extraction–LAMP workflow showing the highest observed detection performance among the three evaluated approaches. The boiling-based and paper-based extraction–LAMP workflows showed lower analytical detection performance and may warrant further investigation as simplified laboratory workflows. These findings require formal analytical validation, reproducibility assessment, and evaluation in larger, independent clinical specimen sets before any clinical, point-of-care, decentralized, or implementation use can be considered.

## Figures and Tables

**Table 1:** Preliminary analytical detection performance of the developed extraction–LAMP workflows evaluated in triplicate, showing the number of positive replicates for each HBV viral load category.

	HBV Viral Load (IU/mL)	10^6^	10^5^	10^4^	10^3^	10^2^	10^1^
Extraction Methods							
Boiling-based		3/3	3/3	0/3	0/3	0/3	0/3
Paper-based		3/3	3/3	0/3	0/3	0/3	0/3
Adapted magnetic bead-based		3/3	3/3	3/3	2/3	0/3	0/3

**Table 2:** Preliminary detection performance of optimized HBV DNA extraction–LAMP workflows compared with real-time PCR.

	Boiling-Based Extraction		Paper-Based Extraction		Adapted Magnetic Bead-Based Extraction	
**Real-time PCR**	Positive *n* (%)	Negative *n* (%)	Positive *n* (%)	Negative *n* (%)	Positive *n* (%)	Negative *n* (%)
Positive (*N* = 35)	15 (43)	20 (57)	19 (54)	16 (46)	22 (63)	13 (37)
Negative (*N* = 5)	0 (0)	5 (100)	0 (0)	5 (100)	0 (0)	5 (100)

*N*, total number of samples; *n*, total number of positive or negative samples.

**Table 3:** Detection rate of optimized HBV extraction–LAMP workflows stratified by HBV viral load.

HBV Viral Load (Log_10_ IU/mL) (*N* = 35)	Total	Boiling-Based Extraction *n* (%)	Paper-Based Extraction *n* (%)	Adapted Magnetic Bead-Based Extraction *n* (%)
7.01–8.00	5	5 (100)	5 (100)	5 (100)
6.01–7.00	5	5 (100)	5 (100)	5 (100)
5.01–6.00	5	4 (80)	5 (100)	5 (100)
4.01–5.00	5	1 (20)	3 (60)	4 (80)
3.01–4.00	5	0 (0)	0 (0)	2 (40)
2.01–3.00	5	0 (0)	1 (20)	1 (20)
<2.00	5	0 (0)	0 (0)	0 (0)
Negative	5	0 (0)	0 (0)	0 (0)

*N*, total number of samples; *n*, total number of positive samples.

## Data Availability

Data are contained within the article or Supplementary Materials.

## Supplementary Materials

The following supporting information can be downloaded at https://www.mdpi.com/article/10.3390/diagnostics16172756/s1; Figure S1: Preliminary analytical detection level of simplified HBV DNA extraction methods combined with colorimetric LAMP; Table S1: Detection performance of four HBV DNA extraction methods combined with colorimetric LAMP according to HBV viral load; Table S2: Detection performance of the developed extraction-LAMP workflows according to clinically relevant HBV DNA levels.
