# Supplementary material for: Pilot Evaluation of Simplified HBV DNA Extraction–LAMP Workflows for Hepatitis B Molecular Testing in Resource-Limited Settings
Source: Diagnostics (Basel). 2026 Aug 28;16(17):2756. doi: 10.3390/diagnostics16172756 (PMC13565630; doi:10.3390/diagnostics16172756)
Supplement: Supplementary file 1 [file diagnostics-16-02756-s001.zip › diagnostics-4438486-supplementary.pdf]

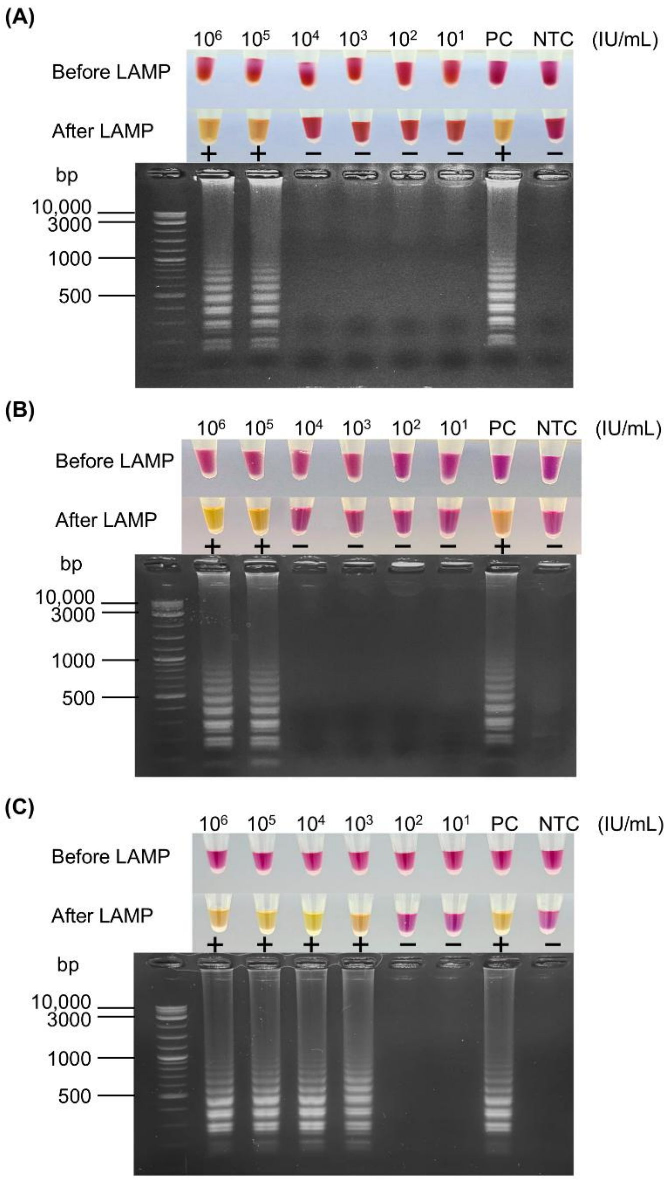

Figure S1. Preliminary analytical detection level of simplified HBV DNA extraction methods combined with colorimetric LAMP. **(A)** Preliminary analytical detection level of the boiling-based extraction-LAMP method. Positive amplification was observed at 10^6 and 10^5 IU/mL, while no amplification was detected at 10^4–10^1 IU/mL, indicating an analytical detection level of 10^5 IU/mL. **(B)** Preliminary analytical detection level of the paper-based extraction-LAMP method. Positive amplification was observed at 10^6 and 10^5 IU/mL, while no amplification was detected at 10^4–10^1 IU/mL, indicating an analytical detection level of 10^5 IU/mL. **(C)** Preliminary analytical detection level of the adapted magnetic bead-based extraction-LAMP method. Positive amplification was observed from 10^6 to 10^3 IU/mL, while no amplification was detected at 10^2 and 10^1 IU/mL, indicating an analytical detection level of 10^3 IU/mL. PC, positive control; NTC, no-template control; IU/mL, international units per milliliter; bp, base pairs.

Table S1. Detection performance of four HBV DNA extraction methods combined with colorimetric LAMP according to HBV viral load.

ID	HBV Viral load (Log_{10} IU/mL)	TIANamp + LAMP	Boiling + LAMP	Paper + LAMP	Magnetic + LAMP
S1	1.11	Negative	Negative	Negative	Negative
S2	1.46	Positive	Negative	Negative	Negative
S3	1.08	Negative	Negative	Negative	Negative
S4	1.18	Negative	Negative	Negative	Negative
S5	1.54	Negative	Negative	Negative	Negative
S6	2.40	Negative	Negative	Negative	Negative
S7	2.50	Negative	Negative	Negative	Negative
S8	2.03	Negative	Negative	Negative	Negative
S9	2.91	Negative	Negative	Positive	Negative
S10	3.00	Positive	Negative	Negative	Positive
S11	3.54	Negative	Negative	Negative	Negative
S12	3.47	Negative	Negative	Negative	Negative
S13	3.81	Positive	Negative	Negative	Positive
S14	3.13	Negative	Negative	Negative	Negative
S15	3.80	Positive	Negative	Negative	Positive
S16	4.75	Positive	Negative	Positive	Positive
S17	4.04	Negative	Negative	Negative	Negative
S18	4.77	Positive	Negative	Negative	Positive
S19	4.82	Positive	Negative	Positive	Positive
S20	4.94	Positive	Positive	Positive	Positive
S21	5.56	Positive	Positive	Positive	Positive
S22	5.52	Positive	Positive	Positive	Positive
S23	5.61	Positive	Positive	Positive	Positive
S24	5.69	Positive	Positive	Positive	Positive
S25	5.54	Positive	Negative	Positive	Positive
S26	6.21	Positive	Positive	Positive	Positive
S27	6.65	Positive	Positive	Positive	Positive
S28	6.28	Positive	Positive	Positive	Positive
S29	6.18	Positive	Positive	Positive	Positive
S30	6.45	Positive	Positive	Positive	Positive
S31	7.23	Positive	Positive	Positive	Positive
S32	7.74	Positive	Positive	Positive	Positive
S33	7.70	Positive	Positive	Positive	Positive
S34	7.68	Positive	Positive	Positive	Positive
S35	7.49	Positive	Positive	Positive	Positive
		n=23	*n*=15	*n*=19	*n*=22

n, total number of positive samples.

Table S2. Detection performance of the developed extraction-LAMP workflows according to clinically relevant HBV DNA levels.

**HBV DNA level (IU/mL)**	**Total**	**Boiling + LAMP** ***n*** **(%)**	**Paper + LAMP** ***n*** **(%)**	**Magnetic + LAMP** ***n*** **(%)**
< 2000	11	0 (0)	1 (9)	1 (9)
2000 – 20,000	5	0 (0)	0 (0)	2 (40)
> 20,000	19	15 (79)	18 (95)	19 (100)

n, total number of positive samples.
